# Author Correction: Rapid multi-directed cholinergic transmission in the central nervous system

**DOI:** 10.1038/s41467-021-22763-3

**Published:** 2021-04-21

**Authors:** Santhosh Sethuramanujam, Akihiro Matsumoto, Geoff deRosenroll, Benjamin Murphy-Baum, Claudio Grosman, J. Michael McIntosh, Miao Jing, Yulong Li, David Berson, Keisuke Yonehara, Gautam B. Awatramani

**Affiliations:** 1 Department of Biology, University of Victoria, Victoria, BC V8W 3N5 Canada; 2 Danish Research Institute of Translational Neuroscience – DANDRITE, Nordic-EMBL Partnership for Molecular Medicine, Department of Biomedicine, Aarhus University, Ole Worms Allé 8, 8000 Aarhus C, Denmark; 3 Department of Molecular and Integrative Physiology, 407 S. Goodwin Ave, Urbana, IL 61801 USA; 4 George E. Whalen Veterans Affairs Medical Center, School of Biological Sciences, University of Utah, Salt Lake City, Utah USA; 5 Department of Psychiatry, School of Biological Sciences, University of Utah, Salt Lake City, UT USA; 6 School of Biological Sciences, University of Utah, Salt Lake City, UT USA; 7 State Key Laboratory of Membrane Biology, Peking University School of Life Sciences, Beijing, 100871 China; 8 Neuroscience, Brown University, 185 Meeting Street, Providence, RI 02912 USA

**Keywords:** Neurotransmitters, Retina

Correction to: *Nature Communications* 10.1038/s41467-021-21680-9, published online 2 March 2021.

The original version of this Article omitted from the author list the 5th author Claudio Grosman, who is from the Department of Molecular and Integrative Physiology, 407 S. Goodwin Ave, Urbana, IL 61801, USA. Additionally, the Author contributions (G.D. and C.G. constructed and tested kinetic nAChR models.) and Acknowledgements (C.G. (R01-NS042169)) were updated to include C.G.’s contribution. This has been corrected in both the PDF and HTML versions of the Article.

Author contributions: S.S. conducted all the electrophysiological and starburst imaging experiments shown in Figs. 2–6. A.M. performed the ACh imaging experiments shown in Fig. 7. Y.L. and M.J. developed ACh sensor; J.M.M. developed the nAChR antagonist; D.B. conducted all the SBEB analysis in Fig. 1; K.Y. designed AAVs constructs and ACh imaging experiments; G.D. and C.G. constructed and tested kinetic nAChR models; B.M.B. wrote new software for image analysis. G.B.A. and S.S. conceived the experiments and wrote the original manuscript.

Santhosh Sethuramanujam^1^, Akihiro Matsumoto^2^, Geoff deRosenroll^1^, Benjamin Murphy-Baum^1^, Claudio Grosman^3^, J. Michael McIntosh^4,5,6^, Miao Jing^7^, Yulong Li^7^, David Berson^8^, Keisuke Yonehara^2*^, and Gautam B. Awatramani^1*^

^1^ Department of Biology, University of Victoria, Victoria, BC V8W 3N5, Canada

^2^ Danish Research Institute of Translational Neuroscience – DANDRITE, Nordic-EMBL Partnership for Molecular Medicine, Department of Biomedicine, Aarhus University, Ole Worms Allé 8, 8000 Aarhus C, Denmark

^3^ Department of Molecular and Integrative Physiology, 407 S. Goodwin Ave, Urbana, IL 61801, USA

^4^ George E. Whalen Veterans Affairs Medical Center, ^5^Department of Psychiatry; ^6^School of Biological Sciences, University of Utah, Salt Lake City, UT, USA

^7^ State Key Laboratory of Membrane Biology, Peking University School of Life Sciences, Beijing 100871, China

^8^ Neuroscience, Brown University, 185 Meeting Street, Providence, RI 02912, USA

