# Supplementary material for: Rapid multi-directed cholinergic transmission in the central nervous system
Source: Nat Commun. 2021 Mar 2;12:1374. doi: 10.1038/s41467-021-21680-9 (PMC7925691; doi:10.1038/s41467-021-21680-9)
Supplement: Supplementary file 1 — Supplementary Information [file 41467_2021_21680_MOESM1_ESM.pdf]

SUPPLEMENTARY FIGURES

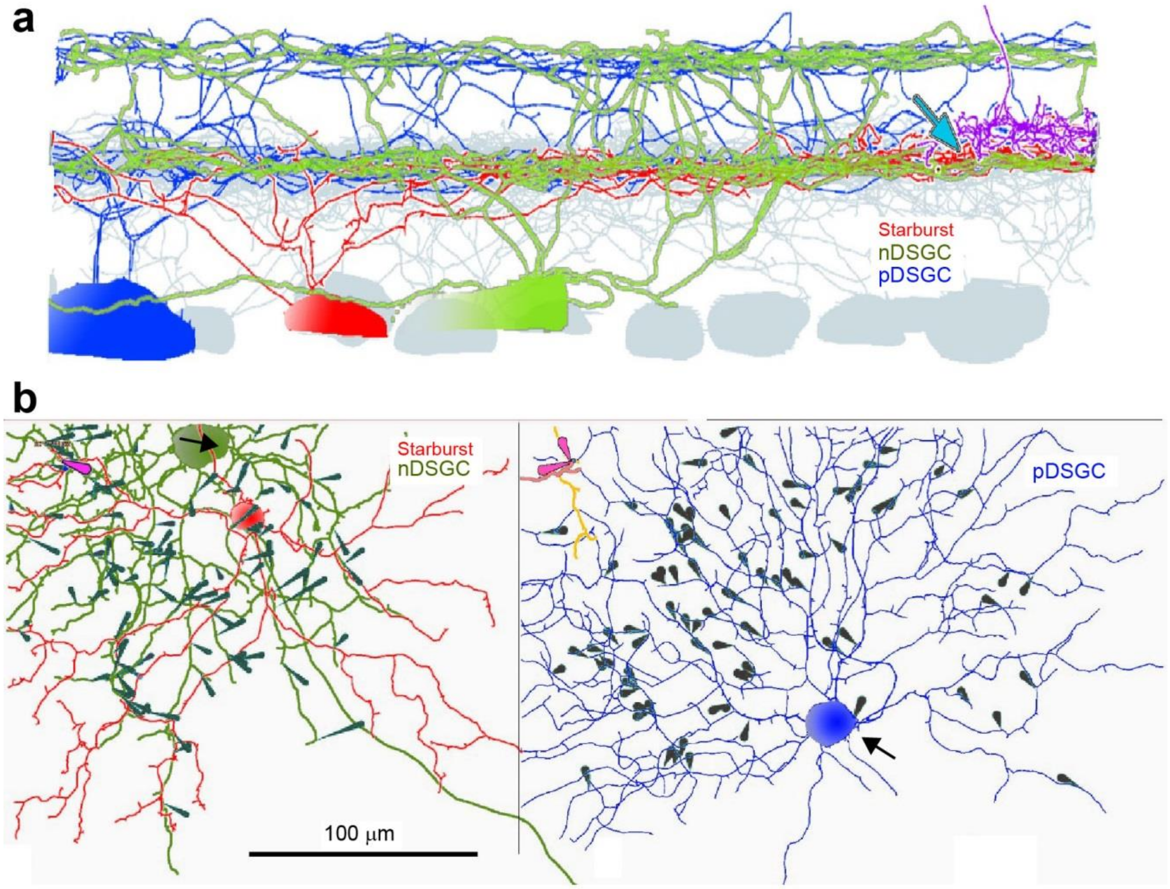

Supplementary Fig. 1: SBEM reconstruction of synapses and contacts between ON starbursts and two postsynaptic ON-OFF DSGCs with opposite preferred directions.
a. Rotated view of the ON starburst (red) and two ON-OFF DSGCs (green and blue) shown in Fig. 1c, overlaid on the profiles of all reconstructed ON starbursts (light grey). The DSGCs show the expected bistratified arbor. The lower (inner) of these two corresponds to the ON starburst plexus. Blue arrow shows the locus of the same synapse shown in Fig. 1c, d. The whole mount view of the ON starburst plexus is shown in Fig. 1a.
b. Opposing patterns of asymmetry in starburst contacts onto the two DSGCs shown in Fig. 1c. Dark green arrows mark the centrifugal orientation of starburst boutons making synaptic contacts onto these two ganglion cells. The green DSGC receives starburst synapses predominantly from processes coursing away from their parent cell body in a leftward and slight upward direction (left). This is true for the synapse from the red starburst as well, whose orientation marker is highlighted in pink. This DSGC thus presumably prefers motion down and right in the volume, as illustrated by the black arrow in the soma. By contrast, the blue DSGC receives starburst synapses coursing in the opposite direction (right), down and right, and thus would be expected to prefer motion up and left in the volume (indicated by arrow close to the soma). The orientation of two starburst processes synapsing onto this cell (peach and gold) is consistent with this, as shown by the pink orientation markers. This analysis to determine the DSGC preferred direction was performed in 6 independent DSGCs, which were used in Fig 1g.

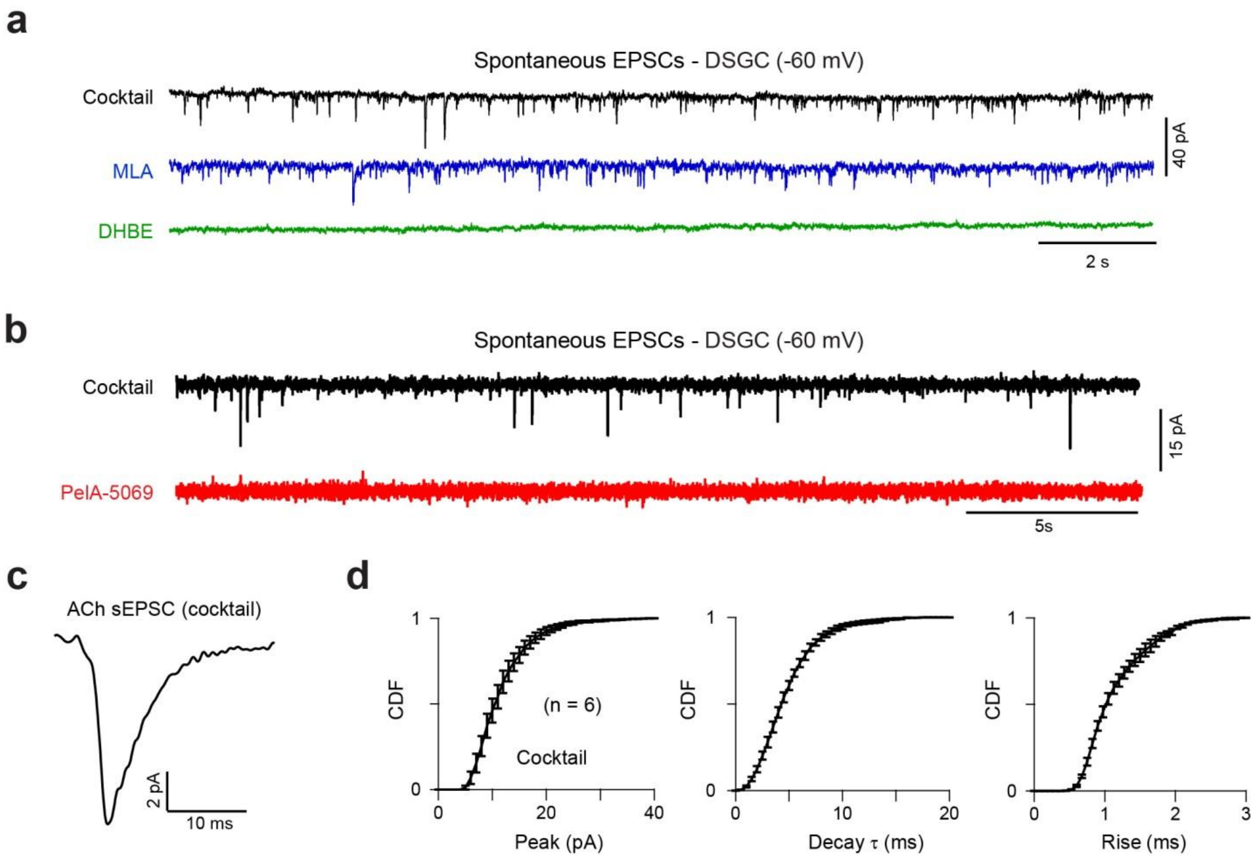

Supplementary Fig. 2: Cholinergic sEPSCs are mediated by α6*-nAChRs

a. Spontaneous cholinergic excitatory postsynaptic currents (sEPSCs) in a DSGC voltage-clamped at -60 mV (E_{Cl}), measured in three different pharmacological conditions. Top trace shows the sEPSCs in the presence of a cocktail blocking glutamatergic transmission with 50 µM DL-AP4, 20 µM CNQX and 100 µM UBP-310. In these experiments, the activity of $GABA_A$ receptors was also blocked with 10 µM SR-95531. Middle and bottom traces show the sEPSCs with the subsequent addition of 10 nM MLA (an α7-nAChR specific antagonist) and 1 µM DHBE (non-α7-nAChR antagonist). DHBE completely blocked the spontaneous events in all 6 DSGCs tested, confirming that the isolated sEPSCs were cholinergic in nature.

b. DSGC sEPSCs measured in the cocktail isolating cholinergic transmission (top) and in the added presence of a peptide shown to specifically block the activity of α6*-subunit containing nAChRs (PelA-5069; bottom). In all the 5 cells tested, sEPSCs were completely blocked by the peptide. Note that the peptide blockade occurred over a slow time course of 15-30 minutes. In these experiments, we controlled for rundown by confirming the presence of sIPSCs, after the cholinergic sEPSCs were completely blocked. PelA-5069 was synthesized as described previously[1].

c. The average trace of the cholinergic sEPSCs observed in the cocktail.

d. A comparison of the cumulative frequency distributions (CDFs) of the peak (left), decay constant (τ) (middle) and rise time (right) of the cholinergic sEPSCs (n = 6 cells; Supplementary Table 1). Data represented as mean ± SEM. Source data are provided as a Source Data file for Supplementary fig. 2a-d.

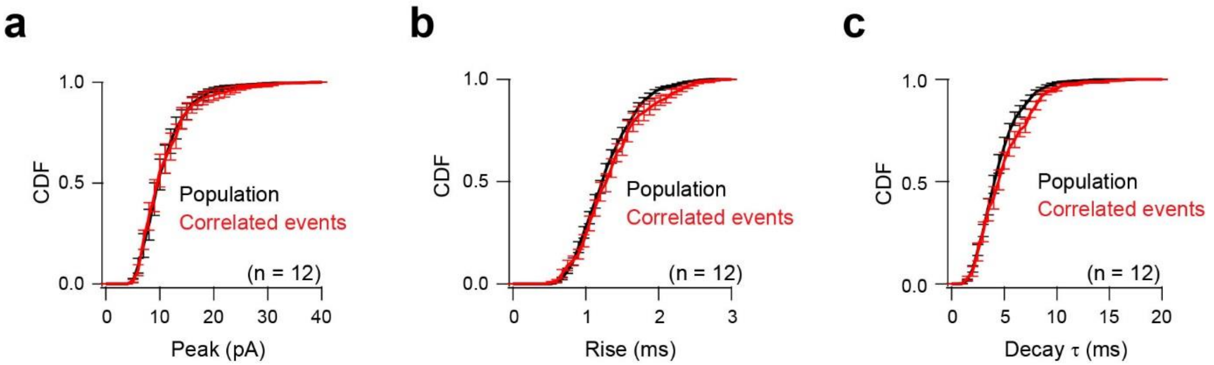

Supplementary Fig. 3: The properties of the correlated sEPSCs are similar to the population distribution.

a. Cumulative frequency distribution (CDF) of the peak amplitude of correlated sEPSCs shown in Fig. 4d compared to the whole sEPSC population (Supplementary Table 1). Spontaneous EPSC data from 12 DSGCs are shown as mean ± SEM.

b, c. Similar to **a**, but for rise times (**b**) and decay τ (**c**) (Supplementary Table 1). Spontaneous EPSC data from 12 DSGCs are shown as mean ± SEM. Source data are provided as a Source Data file for Supplementary fig. 3a-c.

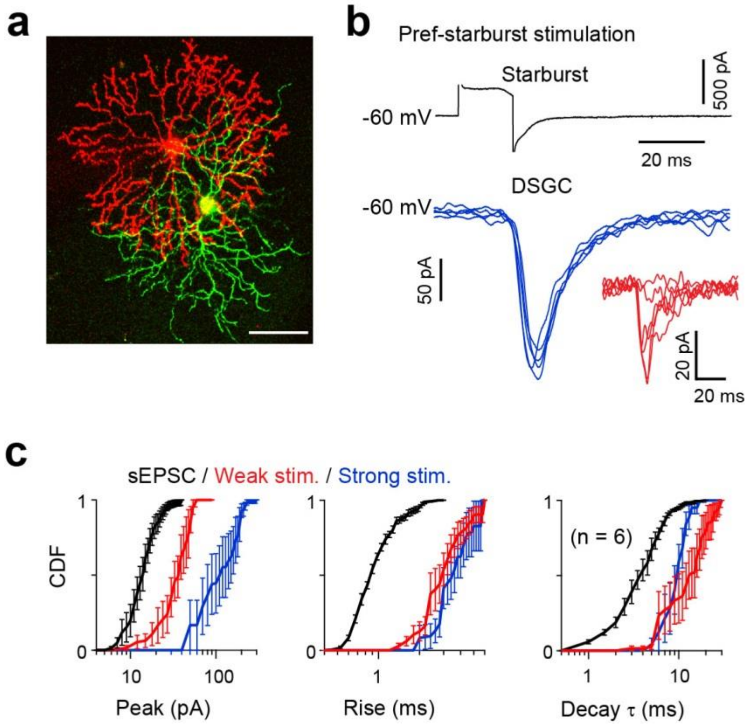

Supplementary Fig. 4: Evoked cholinergic currents are slow to rise relative to spontaneous EPSCs regardless of the level of stimulation, making it difficult to ascertain whether they are mediated by paracrine or synaptic mechanisms.

a. Two-photon image stack showing the morphology of a connected starburst and a DSGC, recovered after a paired whole-cell patch-clamp recording during which they were dialyzed with fluorescent dyes. Scale bar = 50 μm.

b. Brief voltage pulses delivered to the starburst (0 mV, for 17 ms) voltage-clamped at -60 mV, evoked robust cholinergic EPSCs in the DSGC (blue). Weaker pulses (-10 mV, for 17ms) evoked smaller amplitude responses, less reliably (inset; red).

c. A comparison of the mean peak (left), rise time (middle) and decay constant (right) of the sEPSCs with evoked EPSCs (red: weak stimulation; blue: strong stimulation; n = 6 starburst-DSGC pairs from 4 retinas). The kinetics of the evoked EPSCs was slow compared to the sEPSCs. (Supplementary Table 1; peak: $p = 0.0009$; rise: $p = 0.004$; decay: $p = 0.031$; paired t-tests). Data represented as mean ± SEM. Source data are provided as a Source Data file for Supplementary fig. 4b, c.

These experiments illustrate why results from previous studies examining cholinergic connections between starbursts and DSGCs could not be used to infer synaptic or non-synaptic nature of ACh transmission in this circuit.

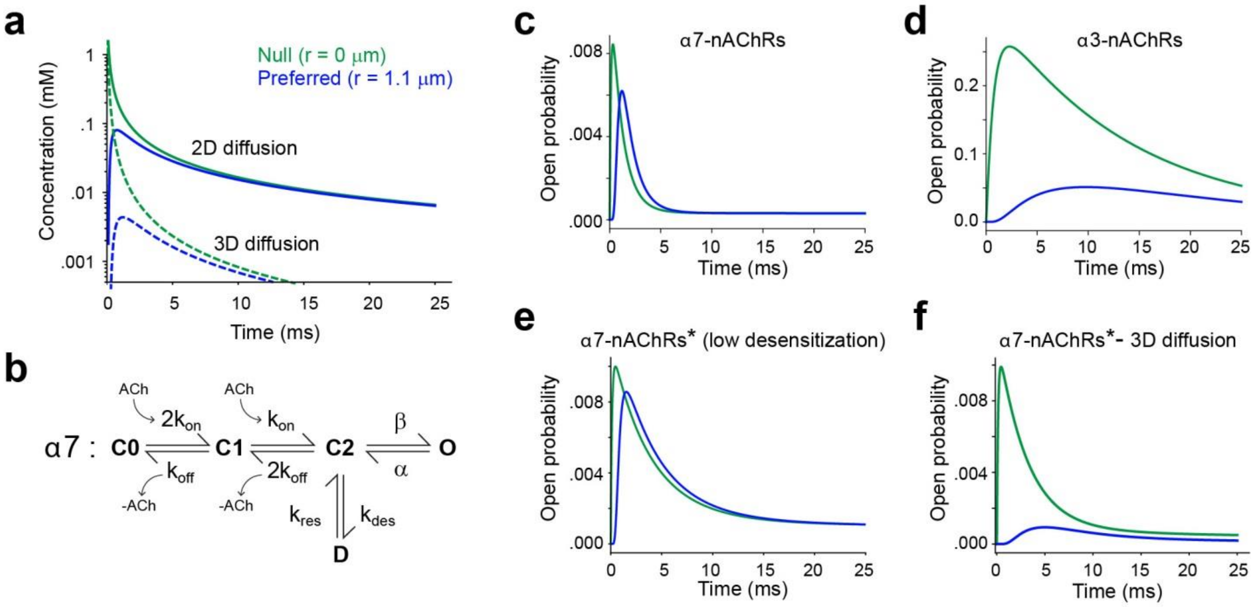

Supplementary Fig. 5: Kinetic model illustrating the potential pre- and postsynaptic factors that promote multi-directed transmission.

a. A plot showing the concentration transients of ACh after a single vesicle release at peripheral 'preferred' sites (r = 1.1μm from the release site; blue) and proximal 'null' sites (r = 0 μm from release site; red) for the cases where ACh spreads in 2D (between pre-and postsynaptic membranes) or in 3D (porous medium), using equations described by Barbour and Hausser[2] (see Supplementary Methods). Note that the concentration gradient for ACh is steeper for the 3-D vs. 2-D diffusion model.

b. Kinetic scheme[3] used to model α7-nAChR mediated responses to brief pulses of ACh shown in **a** (C0: closed, unbound receptor; C1: singly liganded closed receptor; C2: doubly liganded closed receptor; O: receptors in open state; and D: receptors in the desensitized state). A similar scheme was used to model α3-nAChR mediated responses, except the desensitized state (D) was omitted. (See Supplementary Methods for details of the model)

c. Predicted activity at preferred sites (blue) and null sites (red) for 2D diffusion model, when α7-nAChRs are employed. The ratio of peak receptor activity at preferred and null sites was 75%.

d. Similar to **c**, but when α3-nAChRs were employed. The ratio of activity at preferred and null sites was 20%.

e. Similar to **c**, but the desensitization of α7-nAChRs was reduced 4-fold (α7-nAChRs*). In this case, the ratio of preferred-null activity was more than 80%. Subtle differences in amplitude and rise time would be undetectable in our experiments, where somatic whole cell recordings were used to estimate the cholinergic response kinetics.

f. A comparison of the α7-nAChRs* activity at preferred and null sites when the 3D diffusion model was used.

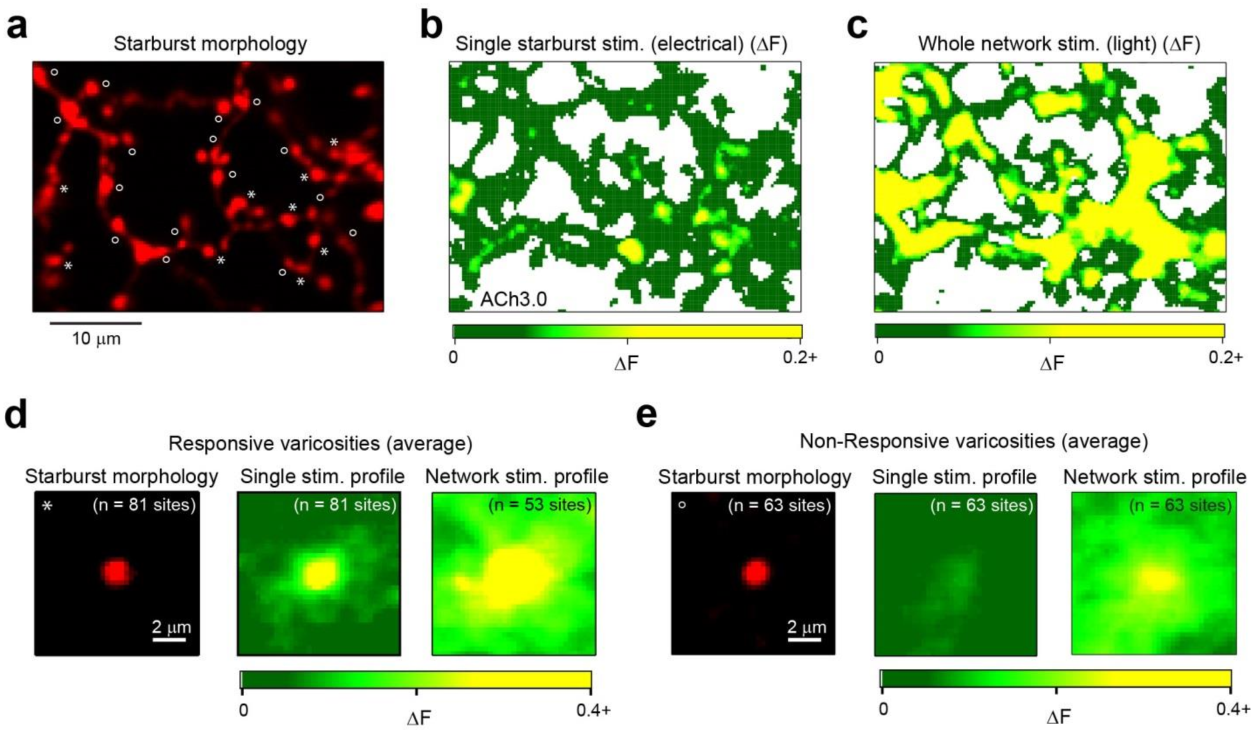

Supplementary Fig. 6: Heterogeneous ACh release properties across starburst varicosities.
a. An image stack showing the morphology of a starburst (red channel) that was loaded with a red dye through the patch electrode used to stimulate it. Markers indicate the heterogeneity in ACh release across starburst varicosities, with * indicating sites which elicited responses upon starburst stimulation, and O indicating sites which did not elicit responses (One such window was recorded in each of the 12 starbursts used in this experiment).
b. An image of the peak ΔF after brief depolarizations of the starburst (single stim.) shown in **a** (average of 7 trials). The white space indicates regions without ACh3.0 expression. A zoomed section of the bottom right is shown in Fig. 6c.
c. Peak change in fluorescence (average of 3 trials) across the same field of view evoked by a moving spot of light. This stimulus activates the whole starburst network resulting in widespread changes in ACh3.0 fluorescence (Whole network stim.).
d. The spatial profile of sites which respond to electrical stimulation of the starburst. The average fluorescence in the red channel (left) is shown weighted to the peak ACh3.0 signals evoked by stimulating single starburst (middle) (n = 81 sites from 12 cells; same as Fig. 6d). The right panel shows the average ACh3.0 signal in a subset of these sites when stimulated with a moving spot of light (n = 53 sites from 7 cells). The ACh sensor signals during light stimulation were widespread in the same regions where signals from electrical stimulation were highly localized. Hence the localized activity observed during electrical stimulation accurately reports on ACh release, and is not an artefact of the sensor expression patterns.
e. Similar to **d**, but for sites which do not respond to electrical stimulation of the starburst (n = 63 sites from 6 cells). Note that these sites responded to stimulating the whole starburst network with light, indicating that the ACh sensor was functional. These observations indicate that these varicosities do not release detectable ACh.

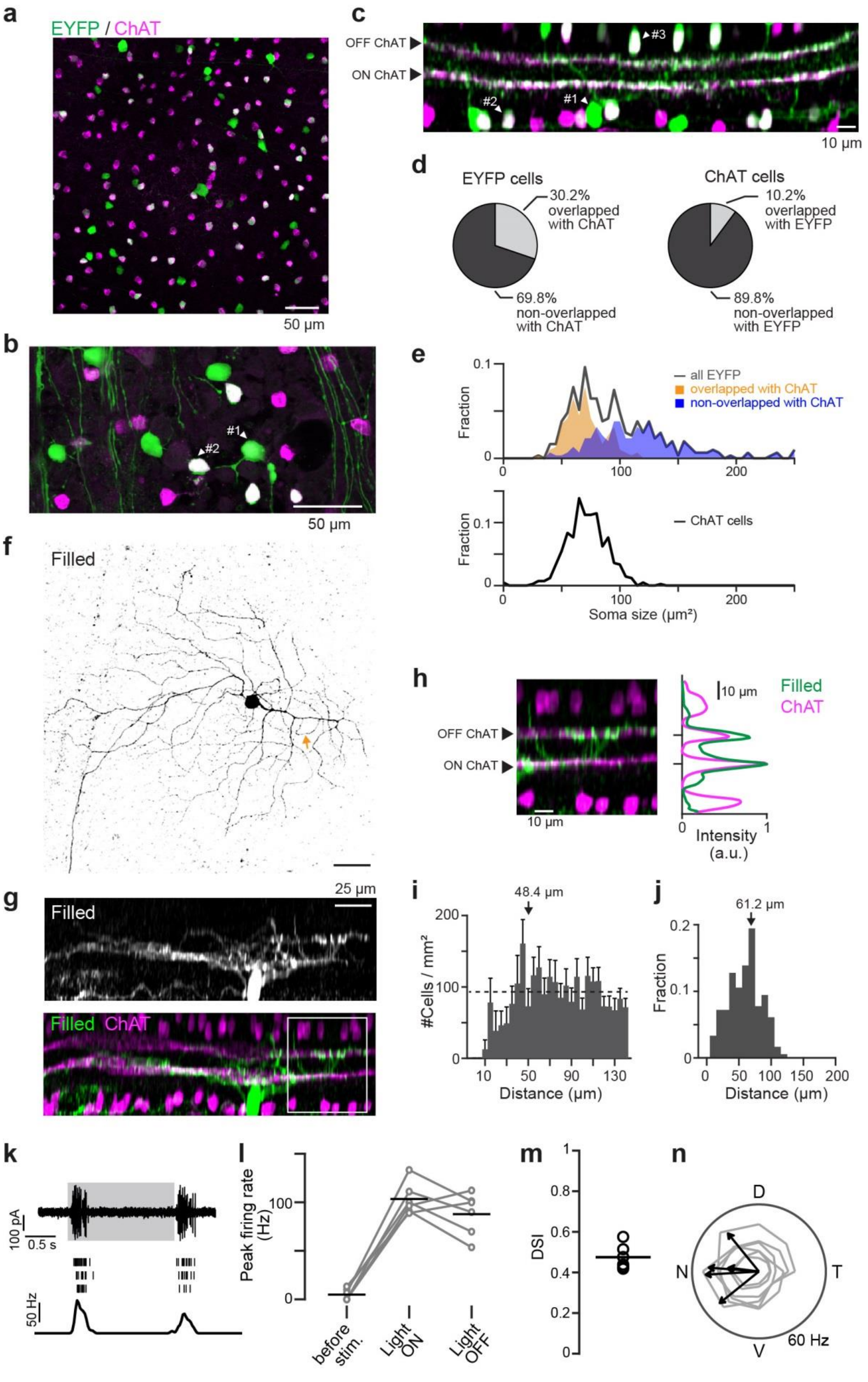

a
EYFP / ChAT
50 µm
b
#1
#2
50 µm
c
OFF ChAT
ON ChAT
#3
#2
#1
10 µm
d
EYFP cells
30.2% overlapped with ChAT
69.8% non-overlapped with ChAT
ChAT cells
10.2% overlapped with EYFP
89.8% non-overlapped with EYFP
e
Fraction
all EYFP
overlapped with ChAT
non-overlapped with ChAT
ChAT cells
Soma size (µm²)
f
Filled
25 µm
g
Filled
Filled ChAT
h
OFF ChAT
ON ChAT
10 µm
10 µm
Filled
ChAT
Intensity (a.u.)
i
48.4 µm
#Cells / mm²
Distance (µm)
j
61.2 µm
Fraction
Distance (µm)
k
100 pA
0.5 s
50 Hz
l
Peak firing rate (Hz)
before stim.
Light ON
Light OFF
m
DSI
n
D
N
T
V
60 Hz

Supplementary Fig. 7: Nasal motion-preferring ON-OFF DSGCs are labeled in Oxtr-T2A-Cre x Thy1-STOP-EYFP mice.

a-c. Example confocal images of a retina in which EYFP (green) and ChAT (magenta; starbursts) signals are labeled in the top view (**a** and **b**) and the side view (**c**). White arrowheads: #1, cell labeled only by EYFP in the ganglion cell layer; #2, cell co-labeled by EYFP and ChAT in the ganglion cell layer; #3, cell co-labeled by EYFP and ChAT in the inner nuclear layer. We examined 8 such FOVs containing 308 EYFP cells and 913 ChAT cells in 3 retinas.

d. Relationship of EYFP-positive and ChAT-positive cells.

e. Histograms of soma size in EYFP cells (top; orange, cells overlapped with ChAT; blue, cells non-overlapped with ChAT) and ChAT cells (bottom). The distribution of soma size of EYFP-ChAT-co-labeled cells (top orange) is similar to that of ChAT cells (bottom).

f-h. Morphology (**f**) and dendritic stratification (**g**) of a single filled Oxtr-cre cell with larger soma. Orange arrow in **f**, an example of a "looping" dendritic arbor (green, filled dendrite; magenta, ChAT). A magnified image of the dendritic stratification (white box in **g**) is shown in **h** (left). Right, the fluorescence intensity profile (green, filled dendrite; magenta, ChAT). This confirmed the observations in **a-c** that the EYFP cells co-stratified with starburst cells, suggesting that these cells were DSGCs. However, this line of analysis was not repeated multiple times, as more direct evidence in the form of their light responses confirmed these EYFP cells to be DSGCs (see **m, n**).

i. Density recovery profile of Oxtr-T2A-Cre labeled cells. Note that the cells overlapped with ChAT were excluded from the calculation. The effective radius was 48.4 um. Data represented as mean ± SD.

j. Histogram of the nearest-neighbor distance in the Oxtr-T2A-Cre labeled cells.

k. Top, example firing responses to a static flash stimulus (gray shaded, 300 µm spot, 2 s duration, 50% contrast) recorded from a labeled cell. Bottom, firing raster in three trials, and a peri-stimulus time histogram.

l. Summary of peak firing rates before stimulus, light ON, and light OFF phases (gray, 6 cells; black, average).

m, n. Directional tunings of the labeled cells (gray, 6 cells; arrow, vector sums in the individual cells) in responses to motion stimulus (**n**; 500 µm in diameter, 1000 µm/s, 50% contrast). The labeled cells showed high direction selective index (DSI; **m**) with an overall preference for nasal direction. Source data are provided as a Source Data file for Supplementary fig. 7e, h, i, j, l, m, n.

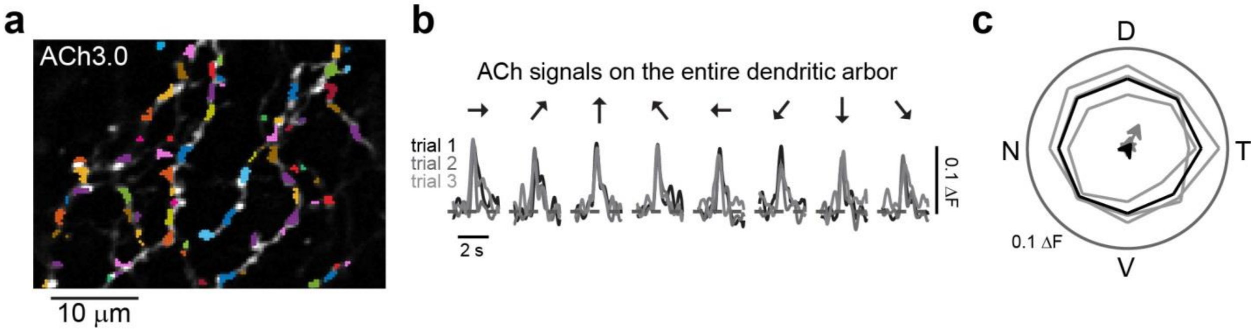

Supplementary Fig. 8: Two-photon acetylcholine imaging with ACh3.0 genetically targeted to nasally-tuned ON-OFF DSGCs.

a. An example field-of-view (FOV) illustrating the dendritic expression of ACh3.0. The pixels with noise correlation higher than 2.5 were used to define regions of interests (ROIs; depicted by the different colors; See Fig. 7a) (see Methods). The detected ROIs with response index higher than 0.6 were classified as 'responsive' (see equation 3). In total, 10 such FOVs were imaged in 3 DSGCs.

b. ACh responses measured across the entire dendritic arbor of the FOV shown in **a** (3 sets of 8 directions). These ΔFs were calculated only from responsive ROIs in the FOV (76 ROIs in **a**).

c. The average response measured in **b** does not show a bias towards the DSGC's preferred direction (i.e. nasal direction). Individual trials are shown in gray and the average is shown in black. Source data are provided as a Source Data file for Supplementary fig. 8c.

SUPPLEMENTARY TABLES

Condition		Peak (pA)	Rise times (ms)	Decay constant (ms)
Fig. 5d, e	Pref. sIPSC (n = 7)	12 ± 1	1.0 ± 0.1	5.4 ± 0.8
	Pref. aIPSC (n = 7)	12 ± 1	1.0 ± 0.1	5.6 ± 0.4
	Null sIPSC (n = 7)	11 ± 1	1.1 ± 0.1	5.1 ± 0.4
	Null aIPSC (n = 7)	11 ± 1	1.1 ± 0.1	4.5 ± 0.6
Supplementary Fig. 2d	Control sEPSC (n = 6)	11 ± 1	1.2 ± 0.1	4.7 ± 0.4
Supplementary Fig. 3	sIPSC whole Population (n = 12)	11 ± 1	1.3 ± 0.1	4.5 ± 0.2
	Correlated sEPSCs only (n = 12)	11 ± 1	1.4 ± 0.1	5.0 ± 0.3
Supplementary Fig. 4c	sEPSC (n = 6)	16 ± 2	0.99 ± 0.03	4.4 ± 0.3
	Weak stim. (n = 6)	38 ± 5	3.3 ± 0.5	15.9 ± 3.8
	Strong stim. (n = 6)	131 ± 25	7.2 ± 1.3	9.8 ± 1.2

Supplementary Table 1: Comparison of spontaneous and evoked response kinetics of cholinergic events under different conditions.

The table shows the peak, rise and decay time constant of cholinergic events under different conditions. Data are represented as mean ± SEM. Source data are provided as a Source Data file.

SUPPLEMENTARY METHODS

In the kinetic model used to estimate cholinergic currents (Supplementary Fig. 5), a series of states were connected by a set of rates that allowed receptors to transition between states. At each time step, the population of receptors that will move from a given state to an adjacent state was calculated by simply multiplying the proportion of receptors in that state with the rate constant and the duration of the time step (1 μs). Each of the rates leaving a given state is applied independently, giving the proportion of receptors that will be transferred to the corresponding adjacent states. Note that moving from unbound to bound states (C0→C1 or C1→C2) is dependent on the concentration of ACh, and hence, in these cases, the transitioning receptor population was calculated by further multiplying rate constants with the ACh concentration. After calculating the transitioning population for each state, the receptor proportions were updated, and this process was repeated for the next time step. At any given time, the proportion of receptors in the open state relative to the total receptor population is used as the model output. The transition rate constants were replicated from Coggan et al.[3] (2005) and are tabulated below.

Receptor transition rate constants	α7-nAChR	α3-nAChR	α7-nAChRs*
k_{on}	$4.1 \times 10^7\ M^{-1}s^{-1}$	$2.3 \times 10^6\ M^{-1}s^{-1}$	$4.1 \times 10^7\ M^{-1}s^{-1}$
k_{off}	82.2/s	84/s	82.2/s
β	86.2/s	513/s	86.2/s
α	7641/s	1000/s	7641/s
k_{des}	879/s	-	220/s
K_{res}	26/s	-	26/s

α7-nAChRs* refers to receptors similar to α7-nAChRs but with a slower desensitization rate. The time course of ACh concentration was modeled using either 2D or 3D diffusion models, replicated from Barbour and Hausser[2] (1997), and described using the following equations.

$$[\mathrm{ACh}](\mathrm{t})_{2\mathrm{D}} = \frac{M}{4h\pi Dt} e^{\left(\frac{-r^2}{4Dt}\right)}$$

$$[\mathrm{ACh}](\mathrm{t})_{3\mathrm{D}} = \frac{M}{8\alpha\left\{\pi\left(\frac{D}{\lambda^2}\right)t\right\}^{3/2}} e^{\left(\frac{-r^2}{4(D/\lambda^2)t}\right)}$$

where $[\mathrm{ACh}](\mathrm{t})_{2\mathrm{D}}$ and $[\mathrm{ACh}](\mathrm{t})_{3\mathrm{D}}$ represents the ACh concentration at the time *t* for the 2D and 3D diffusion models, *M* is the quantity of molecules released (10000), *h* denotes the height of the disc (20nm), *D* is the diffusion coefficient (4×10^{-6} cm^2/s)[4], *r* is the distance from the release site (either 0 or 1.1 was used), α corresponds to the fraction of the extracellular space relative to the total volume (0.21), and λ denotes the tortuosity of the extracellular space (1.55).

To identify the morphology of the labeled EYFP cells in Oxtr-T2A-Cre X Thy1-STOP-EYFP retinas, the cells were filled with neurobiotin. After the recordings, the retinas were fixed for 30 minutes in 4% paraformaldehyde in PBS, and washed with PBS overnight at 4^0C on a shaker. The retinas were incubated in 30% sucrose in PBS for at least 3 hours at room temperature (RT). To enhance the penetration of antibodies, retinas were transferred in the sucrose buffer and

frozen and thawed three times. After washing with PBS, retinas were blocked for 3 hours in blocking buffer (1% bovine serum albumin [BSA], 10% normal donkey serum [NDS], 0.5% TritonX 100, 0.02% sodium azide in PBS) at RT. The retinas were incubated with primary antibodies (chicken anti-GFP 1:1000 [abcam, ab13970]; goat anti-ChAT 1:200 [Milipore, ABN1144P]) for 5 days at RT in antibody reaction buffer (1% BSA, 3% NDS, 0.5% TritonX 100, 0.02% sodium azide in PBS), and secondary antibodies (donkey anti-chicken IgY Alexa 488 1:200 [Jackson ImmunoResearch, AB 2340375]; donkey anti-goat IgG Alexa 568 1:200 [Invitrogen, A11057]; Streptavidin Alexa 633 conjugate 1:200 [ThermoFisher, S21375]) for one day at 4^0C in antibody reaction buffer. After a final washing in PBS, retinas were embedded in Fluoromount-G (eBioscience). The stained retinas were imaged using a confocal microscope (Zeiss LSM 780) using a 40x (1.4 NA) objectives. The images were acquired at 1024 × 1024 pixel (0.35 µm/pixel for 40x), and the optical thickness of each imaging plane in z-stack was 0.3 µm. The images were processed and analyzed using MATLAB. For the morphological characterization of labeled cells, we calculated the distribution of nearest neighbor distances and density recovery profiles (Supplementary Fig. 7). The cells overlapping with ChAT signals were excluded from these calculations.

SUPPLEMENTARY REFERENCES

1 Hone, A. J. *et al.* Positional scanning mutagenesis of alpha-conotoxin PeIA identifies critical residues that confer potency and selectivity for alpha6/alpha3beta2beta3 and alpha3beta2 nicotinic acetylcholine receptors. *The Journal of biological chemistry* **288**, 25428-25439, doi:10.1074/jbc.M113.482059 (2013).

2 Barbour, B. & Hausser, M. Intersynaptic diffusion of neurotransmitter. *Trends in neurosciences* **20**, 377-384, doi:10.1016/s0166-2236(96)20050-5 (1997).

3 Coggan, J. S. *et al.* Evidence for ectopic neurotransmission at a neuronal synapse. *Science* **309**, 446-451, doi:10.1126/science.1108239 (2005).

4 Land, B. R., Harris, W. V., Salpeter, E. E. & Salpeter, M. M. Diffusion and binding constants for acetylcholine derived from the falling phase of miniature endplate currents. *Proceedings of the National Academy of Sciences of the United States of America* **81**, 1594-1598, doi:10.1073/pnas.81.5.1594 (1984).
